# Examination of the Frequency of Soft Tissue Ossification and Calcifications in Panoramic Radiographs: A Retrospective Study

**DOI:** 10.3390/diagnostics15162013

**Published:** 2025-08-12

**Authors:** Sumeyye Celik Ozsoy, Taha Zirek, Serkan Bahrilli, Ibrahim Burak Yuksel, Ali Altindag

**Affiliations:** 1 Department of Oral and Maxillofacial Radiology, Faculty of Dentistry, Karamanoğlu Mehmetbey University, Karaman 70200, Turkey; sumeyyecelik@kmu.edu.tr; 2 Department of Dentomaxillofacial Radiology, Faculty of Dentistry, Necmettin Erbakan University, Konya 42090, Turkey; tahazirek0825@gmail.com (T.Z.); serkanbahrilli@gmail.com (S.B.); alialtindag1412@gmail.com (A.A.)

**Keywords:** head and neck, panoramic radiography, soft tissue calcification

## Abstract

**Background**: This retrospective study aimed to assess the prevalence and distribution of common soft tissue ossifications and calcifications in the head and neck area, such as tonsilloliths, calcified lymph nodes, atherosclerotic plaques, stylohyoid ligament calcifications, and laryngeal cartilage calcifications, using panoramic radiographs (PRs) from a Turkish population. A secondary objective was to analyze these findings based on age and gender, ultimately seeking to enhance clinicians’ awareness of these incidental findings and their potential diagnostic significance. **Methods**: PRs of 1207 patients applying to the Department of Oral and Maxillofacial Radiology at Necmettin Erbakan University Faculty of Dentistry between 2021 and 2022 were reviewed. Out of these, 1193 images meeting quality criteria and showing distinct anatomical details were included. Patients with prior diagnosed bone metabolic disorders were excluded. Two radiologists independently assessed the images for the presence of soft tissue calcifications and ossifications. Inter-observer reliability was quantified using Cohen’s Kappa coefficient, which was found to be 0.78, indicating substantial agreement (95% CI: [0.72–0.83], *p* < 0.001). The calcifications and ossifications were categorized according to age, gender, and type. Data were analyzed employing descriptive statistical methods and Chi-square tests, with a significance level set at *p* < 0.05. **Results**: Soft tissue calcification or ossification was observed in 122 (10.22%) of the 1193 retrospectively evaluated PRs. The most common findings included stylohyoid ligament ossifications (n = 31), laryngeal cartilage calcifications (n = 28), tonsilloliths (n = 25), calcified atherosclerotic plaques (n = 18), and calcified lymph nodes (n = 18). Two antroliths were also identified. Arteriosclerosis, phleboliths, and sialoliths were not detected in this cohort. Although some types of calcification showed numerical variations across age groups and genders (e.g., higher prevalence of most anomalies in patients aged 31 years and older; more frequent laryngeal cartilage calcification in women and tonsilloliths in men), Chi-square analyses revealed no statistically significant association between the presence of these calcifications or ossifications and either age group (*p* = 0.284) or gender (*p* = 0.122). **Conclusions**: PRs serve as an effective initial screening instrument for identifying soft tissue calcifications within the head and neck region, owing to their widespread availability, cost-effectiveness, and minimal radiation exposure. The detection of such findings is of paramount importance, as they may indicate underlying systemic conditions necessitating further diagnostic evaluation. While clinicians should remain vigilant to these anomalies, definitive diagnosis typically requires supplementary imaging modalities such as cone-beam computed tomography (CBCT), ultrasound, or histopathological analysis.

## 1. Introduction

Tissue calcification, a biological process characterized by the deposition of minerals in close proximity to cells, is a fundamental component of normal physiological development in structures such as teeth and bones. However, its occurrence within soft tissues is generally considered an anomalous pathological finding. Research indicates that calcifications across various human tissues often share a convergent developmental pathway, suggesting that inflammatory processes or disturbances in metabolic equilibrium may significantly contribute to their formation [1].

Soft tissue calcifications and ossifications are categorized into distinct groups based on their underlying pathological mechanisms, etiologic factors, and specific anatomical locations [2,3]. Heterotopic ossification refers to conditions where calcium salts accumulate in an organized manner within soft tissues. This phenomenon encompasses three primary forms: styloid ligament ossification, osteoma cutis, and myositis ossificans [2]. Conversely, heterotopic calcification is defined by the disorganized deposition of minerals in soft tissues and is further subdivided into three etiologic categories: dystrophic, metastatic, and idiopathic calcification [4]. In cases of dystrophic calcification, serum calcium and phosphate levels typically remain within normal physiological ranges. These calcifications are commonly observed in diseased, necrotic, or degenerated tissues and are generally believed to arise subsequent to inflammation, infectious diseases, or trauma affecting soft tissues. Examples of dystrophic calcifications include arterial calcifications, cysticercosis, calcified lymph nodes, and tonsilloliths. Metastatic calcifications, however, are characterized by the deposition of minerals in soft tissues due to elevated serum calcium and phosphate levels [2]. These may originate from malignant conditions such as parathyroid carcinoma, multiple myeloma, lymphoma, leukemia, hypopharyngeal squamous cell carcinoma, and breast carcinomas, or from benign etiologies like chronic renal failure, hyperparathyroidism, and hypervitaminosis D [5,6]. Idiopathic calcification occurs when calcium deposits form in soft tissues despite normal serum phosphate and calcium levels, with examples including sialoliths, phleboliths, laryngeal cartilage calcifications, and anthroliths–rhinoliths–dacryoliths [2].

The detection of soft tissue calcifications holds significant clinical relevance, not only for the diagnosis of localized pathologies but also as potential early indicators of systemic diseases. Specifically, the incidental identification of carotid artery calcifications on panoramic radiographs (PRs) in older adults may serve as a valuable prognostic marker for cardiovascular disease risk [7,8]. Beyond systemic implications, PRs are crucial for identifying critical anatomical structures (e.g., precise localization of the mandibular canal for surgical planning) or other unsuspected pathologies, underscoring the comprehensive responsibility of dental practitioners in recognizing all radiographic signs that could signify underlying health conditions [9].

Within the existing literature, most studies investigating soft tissue calcifications utilizing PRs employ a retrospective design and report substantial variations in prevalence across diverse geographic regions and ethnic groups [9,10]. These observed discrepancies are largely attributed to a combination of genetic predispositions and environmental determinants, including dietary practices, smoking habits, medical history of systemic diseases, and age [11]. Consequently, the acquisition of population-specific data, particularly for the Turkish demographic, is paramount for enhancing regional epidemiological awareness. The maxillofacial region comprises a complex array of anatomical structures that exhibit varying radiographic densities, rendering the diagnosis of calcifications originating from pathological conditions challenging, especially in areas immediately adjacent to teeth and bones [12,13]. A PR presents notable advantages as a diagnostic tool due to its low radiation dose, straightforward application, cost-effectiveness, and widespread availability [14]. Soft tissue calcifications, resulting from the accumulation of calcium salts, are typically asymptomatic radiopaque structures frequently identified on PRs during routine dental examinations. Dental clinicians bear the responsibility of accurately identifying these calcifications, differentiating them from anatomical variations, pathological entities, dental anomalies, bone lesions, foreign bodies, artifacts, or normal radiopaque structures, and determining the necessity for further intervention or management [15,16].

In recent years, the landscape of radiographic interpretation has been significantly influenced by advancements in digital imaging and the burgeoning field of artificial intelligence (AI). AI algorithms are increasingly being developed and applied to dental radiographs, offering potential for enhanced diagnostic accuracy, improved detection of subtle anomalies, and streamlined workflow, including the automated identification and classification of soft tissue calcifications [17,18]. Despite these technological innovations, the fundamental importance of accurate human interpretation and a comprehensive understanding of radiographic findings, whether incidental or primary, remains critical for clinical decision-making. Although advanced imaging modalities like cone-beam computed tomography (CBCT) offer superior resolution and three-dimensional assessment, panoramic radiography retains its advantages as a first-line diagnostic tool for asymptomatic individuals due to its extensive anatomical coverage in a single scan and its economic feasibility [17].

Given the clinical significance of these often incidentally discovered soft tissue calcifications and the pressing need for regionally specific epidemiological data, the primary objective of the present study is to ascertain the types and frequencies of soft tissue calcifications incidentally detected during panoramic radiographic examinations within a Turkish population, and to analyze their distribution according to gender and age. We hypothesized that specific patterns in the prevalence and distribution of these calcifications would be discernible across different demographic groups, providing valuable insights for clinical practice.

## 2. Materials and Methods

This retrospective observational study was meticulously carried out at the Department of Oral and Maxillofacial Radiology, Necmettin Erbakan University Faculty of Dentistry. Our research protocol strictly adhered to the ethical guidelines set forth by the Declaration of Helsinki, receiving formal ethical approval from the local Ethics Committee on 25 April 2024, under protocol number 2024/428. Furthermore, the reporting of this study followed the STARD (Standards for Reporting Diagnostic Accuracy Studies) guidelines to ensure comprehensive and transparent presentation of our findings.

### 2.1. Image Acquisition

All PRs drawn upon for this investigation were sourced from the archives of the Oral and Maxillofacial Radiology Clinic at Necmettin Erbakan University Faculty of Dentistry. These particular images were captured between December 2021 and February 2022. The images were consistently obtained using a Planmeca ProOne digital panoramic X-ray device (Planmeca Oy, Helsinki, Finland). To ensure uniformity across the dataset, the manufacturer’s recommended exposure protocols, namely 70 kVp, 8 mA, and 7.4 s, were rigorously applied for all panoramic radiographic exposures. Following image acquisition, a meticulous evaluation process was conducted: all collected data were independently assessed by two experienced oral and maxillofacial radiologists, (T.Z. and S.B.), each with over three years of expertise in dental and maxillofacial radiology. This assessment was performed on an LCD monitor within a controlled ambient lighting environment.

To ensure consistency and reliability, both evaluators underwent a calibration session prior to the study to standardize the diagnostic criteria for identifying and classifying different types of soft tissue calcifications and ossifications. Inter-observer reliability for the identification and classification of soft tissue calcifications and ossifications on PRs was then assessed using Cohen’s Kappa (κ) statistic. The analysis yielded a κ value of 0.78, indicating substantial agreement between the two examiners (95% Confidence Interval: [0.72–0.83], *p* < 0.001), which indicates substantial agreement based on commonly accepted interpretation criteria. Crucially, inter-observer consensus was reached for every type of calcification detected, with each finding then being meticulously documented.

The different types of calcifications were distinguished from overlapping anatomical structures and other radiopacities based on their characteristic radiographic appearance, specific anatomical location, shape, density, and internal structure. For example, tonsilloliths were identified by their multiple, rounded, or oval radiopacities in the tonsillar region, distinct from overlying bone structures. Similarly, calcified atherosclerotic plaques were identified as irregular, nodular radiopacities inferior and posterior to the angle of the mandible, following the course of the carotid artery.

### 2.2. Study Population

From an initial pool of 1207 scanned PRs of patients aged 18 years and above, a substantial subset of 1193 images were ultimately incorporated, having met all the predefined inclusion criteria. Key demographic characteristics of these patients, including age and gender, were systematically registered. For the purpose of analytical clarity, the study population was subsequently stratified into three distinct age brackets: 18–30 years, 31–50 years, and those exceeding 51 years (>51 years).

**Figure 1:**
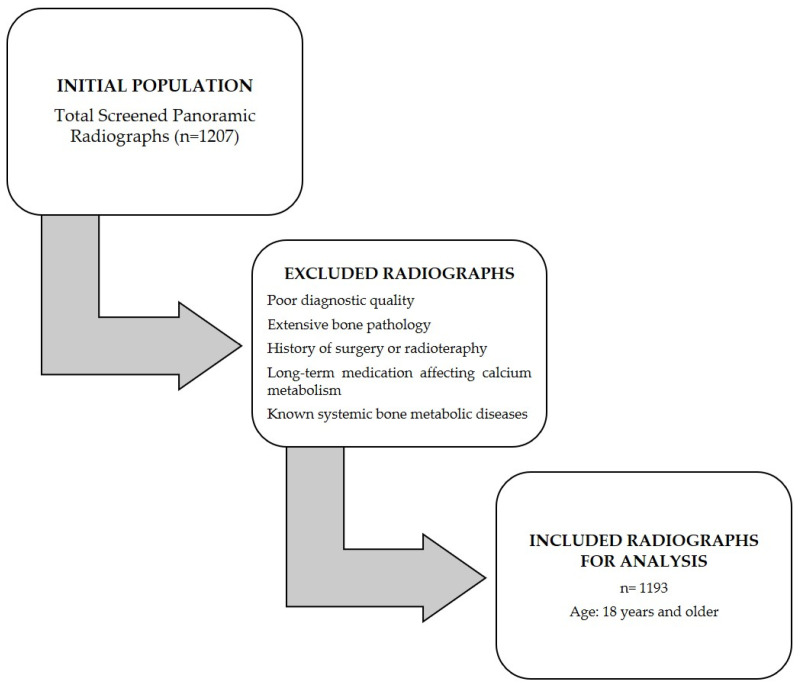
Study flowchart illustrating patient and radiograph selection process.

A flowchart illustrating the selection process of the study population from the initial scanned radiographs, detailing the number of images included and excluded at each stage based on the criteria, is provided in Figure 1.

### 2.3. Inclusion Criteria

- Panoramic radiographs of adequate diagnostic quality (demonstrating optimal contrast, density, and clarity, specifically ensuring complete visualization of critical anatomical regions such as the carotid bifurcation and laryngeal cartilages).
- Images derived from patients presenting without widespread underlying bone pathology (e.g., severe osteoporosis, Paget’s disease, osteomalacia) that could mimic or confound the interpretation of soft tissue calcifications.
- Patients with no documented history of systemic diseases known to significantly impact calcium metabolism (e.g., chronic renal failure, hyperparathyroidism).

### 2.4. Exclusion Criteria

- Images from patients exhibiting extensive bone pathology within the maxillofacial region that could obscure or be confused with soft tissue calcifications.
- Individuals who had undergone resection or cancer surgery in the head and neck area that could alter normal anatomical structures or induce calcification.
- PRs deemed to possess subpar diagnostic quality, exhibiting motion blur, excessive distortion, or containing significant artifacts that could impede accurate interpretation.
- Patients with a prior history of head and neck radiotherapy.
- Patients with documented long-term use of medications affecting calcium metabolism, such as bisphosphonates.
- Absence of corresponding clinical or medical records for correlation, as this study specifically focused on retrospective radiographic interpretation without direct patient follow-up or additional diagnostic validation.

### 2.5. Statistical Analysis

Descriptive statistics were meticulously employed to characterize the study population and delineate the frequency distribution of various soft tissue calcifications and ossifications. The relationship between the incidence of these detected calcifications/ossifications and the demographic variables, specifically age groups and gender, was rigorously assessed utilizing the Chi-square test.

Given the retrospective nature of this study, a pre-hoc power analysis, conventionally conducted to determine the required sample size before data collection, was not applicable. Nevertheless, the substantial sample size of 1193 PRs, as included in our cohort, provided ample statistical power to identify meaningful associations and variations within the population.

All statistical computations were performed using IBM SPSS Statistics software, Version 21.0 (IBM Corp., Armonk, NY, USA). For all analyses, a two-tailed *p*-value of less than 0.05 (*p* < 0.05) was predetermined as the threshold for statistical significance, ensuring a robust interpretation of the findings.

## 3. Results

This comprehensive retrospective evaluation encompassed panoramic radiographic images meticulously acquired from 1193 patients. Within this extensive cohort, soft tissue calcification or ossification was distinctly identified in 122 patients, thereby constituting an overall prevalence rate of 10.22%.

Delving into the demographic profile of these 122 patients in whom calcifications or ossifications were detected, a precise gender distribution emerged: 56 individuals (45.90%) were females, while 66 individuals (54.10%) were males. The mean age for this specific cohort stood at 35.47 years, accompanied by a standard deviation of 8.61 years. A more granular examination of age demographics revealed that the youngest patient exhibiting a detected calcification was 20 years old, whereas the oldest was 76 years. The median age for the entire affected group was determined to be 33 years. Further comprehensive insights into these patients’ ages, encompassing means, medians, and full ranges stratified by gender, are meticulously summarized in Table 1.

The intricate distribution of specific calcification and ossification types across different age groups is explicitly detailed in Table 2. A noteworthy finding was that the predominant proportion of patients presenting with these anomalies fell into the age categories of 31 years and older, collectively accounting for 92 cases (75.41%) of the affected cohort (n = 59 for 31–50 years, n = 33 for 51 + years). Conversely, the 18–30 age group comprised 30 patients (24.59%). When individual calcification types were scrutinized, styloid ligament calcification was observed most frequently in the 31–50 age group (n = 17), while calcified atherosclerotic plaque demonstrated its highest occurrence within the 51+ age group (n = 9). The Chi-square analysis, conducted to ascertain any statistically significant relationship between the presence of these calcifications/ossifications and age groups, yielded a value of χ^2^ = 12.003 with 10 degrees of freedom (d.f = 10), which was not statistically significant (*p* = 0.284).

Table 3 provides a detailed breakdown of calcification and ossification types with respect to patient gender. Numerically, laryngeal cartilage calcification (n = 16) and anthroliths (n = 2) were more frequently identified in women (n = 56), whereas tonsilloliths (n = 18) and calcified lymph nodes (n = 12) showed a higher prevalence among men (n = 66). Notably, styloid ligament ossification (females = 16, males = 15) and calcified atherosclerotic plaques (females = 9, males = 9) exhibited a strikingly even distribution across both genders. A Chi-square test performed to evaluate any statistically significant difference in calcification/ossification distribution based on gender produced a value of χ^2^ = 8.682 with 5 degrees of freedom (d.f = 5), which was also not statistically significant (*p* = 0.122).

**Figure 2:**
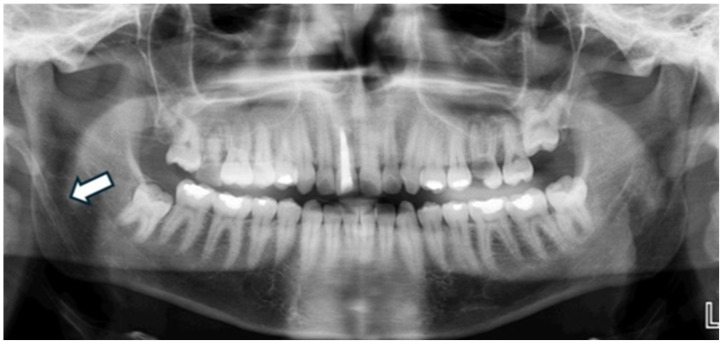
Panoramic radiograph of a 48-year-old male patient demonstrating a unilateral, elongated, and radiopaque structure extending downward from the right temporal region, indicative of styloid ligament calcification.

**Figure 3:**
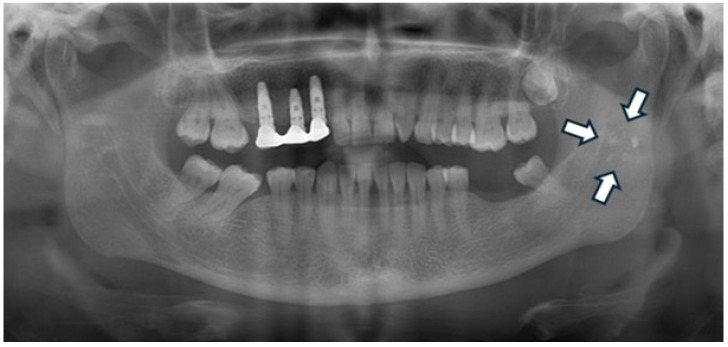
Panoramic radiograph of a 36-year-old male patient showing a well-defined radiopaque mass superimposed over the left mandibular ramus region, consistent with a tonsillolith.

**Figure 4:**
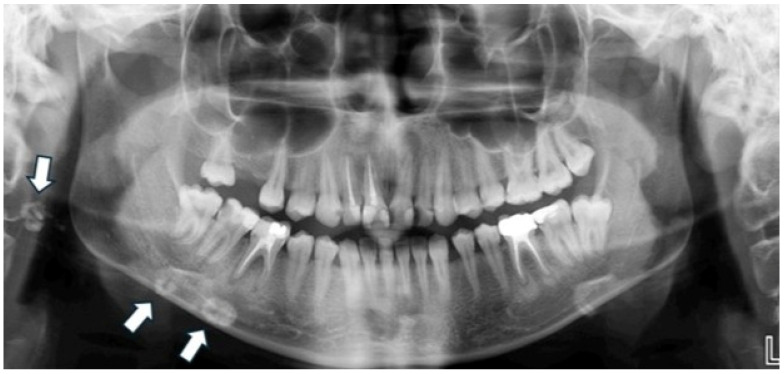
Panoramic radiograph of a 20-year-old female patient showing a unilateral, irregularly shaped radiopaque mass in the right submandibular region, suggestive of a calcified lymph node.

Regarding the overall frequency of each calcification type among the 122 affected patients, styloid ligament ossification emerged as the most prevalent finding, accounting for 31 cases (25.41%) of all detected anomalies (as visually represented in Figure 2). Conversely, anthrolith was the least frequently encountered, with only two cases (1.64%) identified. Other prominent calcifications included laryngeal cartilage calcification (28 cases, 22.95%), tonsillolith (25 cases, 20.49%, illustrated in Figure 3), calcified lymph node (18 cases, 14.75%, depicted in Figure 4), and calcified atherosclerotic plaque (18 cases, 14.75%). It is pertinent to highlight that a thorough investigation for other commonly reported calcifications/ossifications within the head and neck region, such as arteriosclerosis, phleboliths, and sialoliths, yielded no positive findings throughout our study population.

## 4. Discussion

PR, a widely adopted two-dimensional imaging modality in dentistry, consistently furnishes valuable insights into soft tissue calcifications and ossifications within the head and neck region due to its extensive field of view, low radiation dose, and high accessibility [2,18]. While CBCT offers three-dimensional imaging and can be more effective in detecting diminutive and localized calcifications [19,20,21], its interpretation demands enhanced expertise and it possesses certain limitations for soft tissue evaluation. Specifically, CBCT’s shortcomings in soft tissue contrast often render it less optimal for precisely localizing calcifications compared to traditional radiographic methods [22]. Despite these nuances, and the inherent limitations of PR such as geometric distortions and superimpositions [19], PR remains an invaluable and practical first-line diagnostic tool for the preliminary detection and evaluation of various soft tissue calcifications in routine dental practice due to its cost-effectiveness and efficiency [23,24].

This study significantly contributes to the existing body of knowledge by offering comprehensive and contemporary insights into the prevalence and anatomical distribution of various soft tissue calcifications and ossifications within a substantial Turkish population cohort. While numerous prior investigations have explored similar phenomena, our research specifically addresses the pressing need for up-to-date, region-specific epidemiological data. By meticulously analyzing a large sample (n = 1193) and employing rigorous methodology including high inter-observer reliability (κ = 0.78), our work provides a robust dataset from a demographic that has historically been less represented in large-scale panoramic studies. The overall prevalence of soft tissue calcification/ossification in our study was 10.22% (122 out of 1193 patients). This rate, while at the upper end, aligns with the broad range (typically 2.61% to 8%) reported in the existing literature [24,25,26]. However, it starkly contrasts with studies reporting significantly higher prevalences, such as Acıkgöz and Akkemik [9], who found a 35.8% prevalence in another Turkish population (9553 patients). These substantial variations across different studies and populations, even within the same ethnic group, highlight the complex interplay of factors including ethnic and genetic predispositions, distinct dietary habits, varied imaging protocols (e.g., specific panoramic device models and exposure parameters which may influence sensitivity to subtle calcifications), environmental factors (e.g., fluoride levels), and differences in observer experience and diagnostic criteria [27]. Our detailed analysis, therefore, not only adds valuable regional epidemiological data but also underscores the variability inherent in such findings, emphasizing the need for standardized reporting.

Demographic characteristics, particularly age and gender, are commonly investigated factors influencing the prevalence and distribution of soft tissue calcifications. The literature presents conflicting accounts regarding gender predilection; some studies indicate a higher prevalence in women [14,24], others in men [20,28], while a third group finds no appreciable gender difference [25,29]. In our current investigation, while the numerical incidence of overall calcification/ossification was slightly higher in men (54.10% of the affected cohort), and certain calcification types showed numerical variations across age groups (e.g., 75.41% of detected calcifications in individuals aged over 30 years, aligning with observations from Aragoneses et al. [30]), Chi-square analyses revealed no statistically significant association between the presence of these calcifications/ossifications and either age group (*p* = 0.284) or gender (*p* = 0.122).

The absence of statistical significance, despite observable numerical trends, underscores the complex and multifactorial etiology of soft tissue calcifications. These calcifications may not consistently demonstrate clear demographic correlations within specific populations or when examined solely through panoramic radiography. It is inferred that age-related degenerative, inflammatory, and metabolic changes likely contribute to soft tissue calcification, as supported by prior research. However, these broad demographic factors may not always attain statistical significance across all cohorts, potentially due to limitations such as sample size, unique population characteristics, or the inherent two-dimensional nature of panoramic imaging, which can obscure subtle correlations. Nonetheless, our findings continue to highlight the importance of including individuals across all age groups in radiographic screening strategies, as calcifications were detected in individuals aged from 20 to 76 years.

The head and neck region is frequently host to various soft tissue calcifications and ossifications, including tonsilloliths, stylohyoid ligament calcifications, lymph node calcifications, carotid artery calcifications, and laryngeal cartilage calcifications [19]. In our study, we successfully detected stylohyoid ligament ossification (n = 31), laryngeal cartilage calcification (n = 28), tonsillolith (n = 25), calcified lymph node (n = 18), calcified atherosclerotic plaque (n = 18), and anthroliths (n = 2). Conversely, a thorough search failed to yield findings for arteriosclerosis, phleboliths, and sialoliths within our cohort.

The absence of certain commonly reported calcifications, such as sialoliths and phleboliths, in our population, presents a notable contrast to some previous large-scale studies [9,25]. For example, Acikgoz and Akkemik [9] reported a broader spectrum of calcifications, including rarer types, within another Turkish population. This observed discrepancy underscores the potential influence of various factors on detection rates, including differences in sample size, image resolution and quality (e.g., specific parameters of different panoramic devices), and the specific radiographic expertise of the observers. While our study aimed for comprehensive detection and employed rigorous inter-observer agreement protocols, these variations highlight that panoramic radiography, despite its screening utility, may not always capture all subtle or nascent calcifications that could be visible with advanced three-dimensional imaging modalities like CBCT or through clinical examination. Therefore, the non-detection of these specific types does not definitively rule out their presence in the population but rather reflects the limitations inherent to the retrospective 2D radiographic assessment in our study. This further reinforces the need for future comparative studies that leverage multiple imaging modalities for a more comprehensive assessment.

Stylohyoid ligament ossification proved to be the most common type of calcification/ossification in our study, registering 31 cases, representing 25.41% of all detected anomalies. The styloid process (SP), an approximate 2.5 cm long, thin, cylindrical bony extension projecting downwards from the temporal bone’s pars tympanica, is anatomically situated between the carotid arteries and the internal jugular vein, anteromedial to the stylomastoid foramen and posterior to the tonsillar fossa. The “stylohyoid complex” refers to the anatomical unit formed by the SP, the stylohyoid ligament, and the lesser horn of the hyoid bone.

While the existing literature often suggests a higher prevalence of stylohyoid ligament ossification in women than men [31], with some researchers linking higher symptom rates and greater occurrence in older women to potential menopausal changes [32], our study yielded a noteworthy and divergent outcome. Contrary to these reports, stylohyoid ligament ossification was found to be nearly equally distributed between men (n = 15) and women (n = 16) in our specific Turkish cohort. This finding, which deviates from some commonly accepted gender predilections, highlights the importance of population-specific epidemiological studies. It suggests that demographic patterns in stylohyoid ligament ossification prevalence may not be universal and could be influenced by a complex interplay of genetic, environmental, or lifestyle factors unique to different populations, warranting further investigation.

Laryngeal cartilage calcification constituted 28 cases, representing 22.95% of our findings. The calcified triticeous cartilage, commonly observed beneath the greater horn of the hyoid bone and near the superior border of the C4 vertebra on panoramic and lateral head radiographs, is a prominent example. Another frequently encountered laryngeal cartilage calcification on PRs, the upper horn of the calcified thyroid cartilage, is situated medial to the fourth cervical vertebra and often superimposes onto the prevertebral soft tissue [2]. The literature generally reports its incidence as 8.6–10.25%, often with a higher predilection for women [33,34].

Our study’s observation that laryngeal cartilage calcification was more common in women (n = 16 compared to n = 12 in men) aligns with the existing literature regarding gender distribution, reinforcing a consistent finding across various populations. However, our detected frequency of 22.95% appears numerically higher than the typically reported rates (8.6–10.25%). This noteworthy difference in prevalence might be attributable to a combination of population-specific factors (e.g., genetic predispositions, environmental influences, average age of the cohort) and diagnostic methodologies employed (e.g., image quality, specific panoramic device characteristics, or observer experience and calibration, as discussed previously). Our findings, therefore, contribute to a more nuanced understanding of the true prevalence of laryngeal cartilage calcification in diverse populations.

Beyond specific calcification types, our study’s observed gender distribution patterns for tonsilloliths and laryngeal cartilage calcifications notably diverge from several previous investigations. While earlier studies often reported a significant male predominance for tonsilloliths, our data suggest a more balanced gender ratio for this type of calcification (n = 7 females, n = 18 males). This deviation from established trends highlights the dynamic nature of epidemiological findings across diverse populations. Such evolving patterns may well be influenced by changing lifestyle factors, dietary habits, and the prevalence of specific comorbidities within populations, thereby reinforcing the pressing need for re-evaluating diagnostic assumptions in dental radiology. This perspective finds strong support in Davenport (2023), who critically discussed the profound ways in which incidental imaging findings influence downstream clinical decisions and patient demographics [34,35].

Dystrophic calcification, stemming from chronic tonsil inflammation, is termed tonsillolith. On panoramic imaging, it typically manifests as one or several small radiopacities superimposed on the middle portion of the ramus height [36]. Reported frequencies of tonsilloliths on PRs span a range between 1.45% and 8.14% [24,37]. In our investigation, we identified tonsilloliths in 25 cases, representing a prevalence of 20.49%. This rate is notably higher than figures commonly reported in the literature, which typically fall within the 1.45% to 8.14% range. This significant disparity warrants further exploration and highlights the potential for population-specific variations, differences in diagnostic criteria, or improved detection capabilities in our study cohort.

Calcified lymph nodes, one of the most commonly observed soft tissue calcifications, arise as a sequela of chronic inflammation within the lymph nodes, particularly due to granulomatous diseases. The submandibular region represents the most frequent site of observation on panoramic radiography [2]. In a study by Eisenkraft and Som [38], which meticulously examined cervical lymph nodes in 2300 CT images, calcified lymph nodes were detected at a rate of 1%. In our study, we identified 18 cases (14.75%) of calcified lymph nodes. While this incidence rate is numerically higher than the 1% reported in the CT-based study by Eisenkraft and Som, this difference is likely attributable to the distinct nature of the imaging modalities (2D panoramic vs. 3D CT) and their respective patient populations. Despite the numerical variance, our finding reinforces the understanding that calcified lymph nodes are a common incidental finding on PRs, emphasizing their frequent occurrence in routine dental screenings.

Calcified atherosclerotic plaque, resulting from the calcification of atherosclerotic changes within the intima layer of affected vessels, holds substantial clinical significance as it can lead to cerebrovascular and embolic diseases. Therefore, it is of utmost importance that patients whose panoramic radiographs display calcification consistent with an atherosclerotic plaque be promptly referred to a specialist for further comprehensive examinations pertaining to cerebrovascular and cardiovascular diseases [39].

Bayer et al. [40], in their study involving 2557 PRs, reported an incidence of calcified atherosclerotic plaque at 4.8%. Likewise, Atalay et al. [41] found a rate of 5.63% in their examination of 1650 PRs. In our study, the detected prevalence of calcified atherosclerotic plaques was 18 cases (14.75%). This rate is notably higher compared to some similar panoramic radiography studies, such as those by Bayer et al. and Atalay et al. This elevated detection rate in our cohort may be attributed to several factors, including differences in population health profiles, image resolution, and diagnostic criteria. For instance, Janiszewska-Olszowska et al. [42] reported indeed higher detection rates, underscoring that meticulous radiographic technique and effective cardiovascular risk stratification can significantly influence diagnostic outcomes. These findings strongly reinforce the inherent value of PRs as an effective preliminary screening tool for identifying vascular calcifications, allowing for early referral and potential intervention in asymptomatic individuals [5].

Consistent with our findings, Sisman et al. [43], in their study examining 750 panoramic radiographs to ascertain the incidence of calcified atherosclerotic plaque in the Cappadocia region, found no significant difference between women and men, echoing the balanced distribution (females = 9, males = 9) observed in our current study.

Recent research highlights the rapidly evolving role of AI in diagnostic radiology. AI-supported panoramic readings hold significant promise for enhancing the diagnostic accuracy of detecting various soft tissue calcifications, particularly vascular calcifications like carotid calcifications, where some studies report improved sensitivity [40]. These findings underscore the considerable potential of AI algorithms in augmenting traditional image interpretation by boosting detection capabilities, offering the promise of improved efficiency and earlier identification of clinically significant anomalies.

However, the application of AI in this domain is not without its challenges. The observed variation in AI performance across different calcification types—for instance, where sensitivity remains a challenge for identifying lymph node or sialolith calcifications (specificity range: 0.17–1.00) [40]—highlights the imperative need for further refinement and extensive training of AI systems. This is particularly crucial for the subtle differentiation of nuanced or overlapping radiopacities. As AI becomes increasingly integrated into dental diagnostics, standardizing its applications and rigorously validating its results across diverse populations will prove essential for reliable clinical adoption. Similar conclusions were drawn by Zadrożny et al. [44], who evaluated an AI-based PR analysis system and reported high specificity (>0.9) and reproducibility (ICC > 0.75) across multiple dental features, albeit with performance variations contingent on the specific pathology.

While AI tools were not directly applied in our current study, our meticulous manual identification and categorization of diverse soft tissue calcifications on panoramic radiographs undeniably underscore a compelling opportunity for future AI model development and validation in this domain. Our comprehensive dataset, particularly given its focus on a Turkish population, can serve as valuable ground truth data for training and testing next-generation AI algorithms, ultimately contributing to more accurate and efficient radiographic diagnoses in routine clinical practice.

Unlike many investigations that tend to narrow their focus to specific subtypes of calcifications, our current study encompasses a broad spectrum of ossifications and calcifications, thereby offering a more comprehensive epidemiological picture within the Turkish population. This inclusive methodological approach is deemed essential for capturing relatively rare entities like anthroliths (n = 2), which are frequently omitted from more narrowly scoped studies. İspir et al. [45] emphasized the profound value of CBCT in identifying incidental soft tissue calcifications and advocated for such broad evaluations to significantly enhance diagnostic accuracy, a principle reinforced by our findings on the diverse range of calcifications detectable with panoramic radiography.

### Limitations

Our study, like all research, has limitations that deserve careful consideration. First, the single-institution, cross-sectional design naturally limits the generalizability of our results, as risk factors for soft tissue ossification may differ across various regions and populations. Second, and most importantly, the lack of comprehensive clinical evaluation and follow-up data in our retrospective approach is a major limitation. This restricted the depth of our analysis, preventing us from establishing direct and definitive links between radiographic findings and patient symptoms or underlying systemic conditions. As a result, although we detected various calcifications, this study could not determine the clinical significance of these findings or distinguish incidental findings from those with immediate pathological implications based solely on PRs. For instance, while carotid artery calcifications are known radiographic markers, their exact clinical importance for individual patients requires further medical investigation. Finally, it is possible that some calcifications, despite existing, went undetected due to the inherent limitations of panoramic radiography, especially issues related to superimposition and two-dimensional imaging. Comparing these findings with more advanced imaging methods like CBCT or ultrasound, for definitive diagnosis, was outside the scope of this study.

## 5. Conclusions

In summation, PRs unequivocally prove to be an effective and invaluable initial screening tool for the incidental detection of a wide array of soft tissue calcifications and ossifications within the head and neck region. While our study, conducted within a Turkish population, did not find a statistically significant association between the presence of these calcifications and patient age or gender, our findings provide crucial population-specific prevalence data and highlight numerical trends in their distribution. The incidental detection of these entities by dental practitioners can serve as a crucial early indicator for potential underlying systemic conditions, thereby highlighting the pivotal and expanding role of dentists in delivering comprehensive patient care and facilitating timely medical referrals when clinically warranted. However, it is important to remember that definitive diagnosis often necessitates further advanced imaging or clinical correlation.

This study was previously presented in part as a conference abstract and published in the proceedings of the 3rd International Dentistry Congress, held in Konya, Türkiye, on 24–26 May 2024 [46].

## Figures and Tables

**Table 1:** Descriptive statistics for patients’ age.

	Female	Male	Total
Number	56	66	122
Average	35.13	35.60	35.47
Standard Deviation	8.21	8.92	8.61
Median	33	34	33
Smallest Value	20	20	20
Greatest Value	74	76	76

**Table 2:** Cross-tabulation and Chi-square test for calcification types and age groups.

Calcification/Ossification Types							
Age Groups	Styloid Ligament Calcification	Laryngeal Cartile Calcification	Tonsilolith	Calcified Lymph Node	Calcified Atherosclerotic Plaque	Anthrolith	Total
18–30	11	7	5	4	2	1	30
31–50	17	13	12	9	7	1	59
51+	3	8	8	5	9	0	33
Total	31	28	25	18	18	2	122

χ^2^ = 12.003, d.f. = 10, *p* = 0.284.

**Table 3:** Cross-tabulation and Chi-square test for calcification types and gender.

Calcification/Ossification Types							
Gender	Styloid Ligament Calcification	Laryngeal Cartile Calcification	Tonsillolith	Calcified Lymph Node	Calcified Atherosclerotic Plaque	Anthrolith	Total
Female	16	16	7	6	9	2	56
Male	15	12	18	12	9	0	66
Total	31	28	25	18	18	2	122

χ^2^ = 8.682, d.f. = 5, *p* = 0.122.

## Data Availability

The datasets can be shared with researchers who wish to conduct studies upon reasonable request.
